# Impact of heat on metabolic and hemodynamic changes in transcranial infrared laser stimulation measured by broadband near-infrared spectroscopy

**DOI:** 10.1117/1.NPh.5.1.011004

**Published:** 2017-09-19

**Authors:** Xinlong Wang, Divya D. Reddy, Sahil S. Nalawade, Suvra Pal, F. Gonzalez-Lima, Hanli Liu

**Affiliations:** a University of Texas at Arlington, Department of Bioengineering, Arlington, Texas, United States; b University of Texas at Arlington, Department of Mathematics, Arlington, Texas, United States; c University of Texas at Austin, Department of Psychology and Institute for Neuroscience, Austin, Texas, United States

**Keywords:** transcranial infrared laser stimulation, photobiomodulation, near-infrared spectroscopy, heat

## Abstract

Transcranial infrared laser stimulation (TILS) has shown effectiveness in improving human cognition and was investigated using broadband near-infrared spectroscopy (bb-NIRS) in our previous study, but the effect of laser heating on the actual bb-NIRS measurements was not investigated. To address this potential confounding factor, 11 human participants were studied. First, we measured time-dependent temperature increases on forehead skin using clinical-grade thermometers following the TILS experimental protocol used in our previous study. Second, a subject-averaged, time-dependent temperature alteration curve was obtained, based on which a heat generator was controlled to induce the same temperature increase at the same forehead location that TILS was delivered on each participant. Third, the same bb-NIRS system was employed to monitor hemodynamic and metabolic changes of forehead tissue near the thermal stimulation site before, during, and after the heat stimulation. The results showed that cytochrome-c-oxidase of forehead tissue was not significantly modified by this heat stimulation. Significant differences in oxyhemoglobin, total hemoglobin, and differential hemoglobin concentrations were observed during the heat stimulation period versus the laser stimulation. The study demonstrated a transient hemodynamic effect of heat-based stimulation distinct to that of TILS. We concluded that the observed effects of TILS on cerebral hemodynamics and metabolism are not induced by heating the skin.

## 1 Introduction

The concept of using near-infrared or infrared light to modulate biological functions, also known as photobiomodulation (PBM), has recently gained rising attention since it may serve as an effective, noninvasive, interventional tool for multiple neural applications in the future.^1^^–^^4^ For example, transcranial infrared laser stimulation (TILS) with 1064-nm laser applied to the forehead has served as a particular approach of brain PBM for improving human neurocognitive functions, such as attention, memory, and executive functions.^4^^–^^8^ A couple of mechanistic studies on TILS were recently reported by Wang et al.,^9^^,^^10^ supporting the hypothesis that photons at 1064 nm oxidize cytochrome c oxidase (CCO), the terminal enzyme in the mitochondrial respiratory chain. Light absorption of CCO^11^ effectively contributes to oxygen and energy metabolism in neurons.^12^ TILS leads to upregulation of cerebral CCO and hemodynamics as well as increases in cerebral oxygen consumption.^9^^,^^10^^,^^13^ The mechanism of TILS supported/discussed in Refs. 9 and 10 helps us understand the relationship between metabolic and hemodynamic changes,^14^ and provides a mechanistic explanation for beneficial neural effects of PBM and/or TILS in a number of medical conditions.^5^^–^^8^

However, besides metabolic and hemodynamic effects on cerebral tissues, TILS may generate non-negligible thermal effects that may confound the results of previous studies^9^^,^^10^ due to laser heating on the tissue. Up to now, while several beneficial effects of PBM have been reported for its therapeutic use,^15^^,^^16^ the contribution of heat generated from near-infrared or infrared light toward any of the studied positive effects has never been tested. In principle, continuous irradiance with laser or light-emitting diodes over a period of time at a particular region of interest would result in an accumulated thermal effect and thus lead to an increase of skin or local temperature at the stimulated region. Such a thermal effect could lead to local increases of blood flow and tissue oxygenation, which could confound the association or interplay between metabolic and hemodynamic effects induced by TILS.^5^^,^^6^ Specifically, it was unclear whether our measured changes of oxyhemoglobin, deoxyhemoglobin, and total-hemoglobin concentration (i.e., Δ[HbO], Δ[HHb], and Δ[HbT]) under TILS were induced by the enhanced metabolism (i.e., increased oxidized CCO concentration, Δ[CCO]) or by the TILS-produced thermal effect. The objective of this study was to quantitatively assess TILS-induced thermal effects on metabolic and hemodynamic changes of forehead tissue measured by broadband near-infrared spectroscopy (bb-NIRS), as well as to confirm/demonstrate the potential role of such thermal effects on hemodynamic changes of forehead tissue determined by bb-NIRS.

## 2 Materials and Methods

### 2.1 Brief Review of Previous TILS Setup and Measurements

We recently reported that TILS could result in upregulation of cerebral CCO and hemodynamics as well as increases in cerebral oxygen consumption.^9^^,^^10^^,^^13^ While details on TILS setup and experimental protocols were given in Refs. 9, 10, and 13, we briefly review related information on the TILS experimental setup and protocols here for the reader’s convenience.

The laser used in our previous TILS studies was a 1064-nm continuous wave laser device (HD Laser Model CG-5000, Cell Gen Therapeutics LLC, Dallas, Texas), which has been Food and Drug Administration (FDA) cleared for various uses in humans.^17^^,^^18^ The laser light was delivered from a handpiece with a beam area of $13.6\;{\mathrm{cm}}^{2}$. Since the laser was collimated, the laser beam’s size was kept approximately the same from the laser aperture to the stimulation spot on the participant’s forehead. The laser power during TILS was kept ∼3.4  W with a power density in the beam area of $0.25\;W/{\mathrm{cm}}^{2}$, the same as that reported in previous studies.^4^^,^^5^^,^^9^^,^^10^^,^^13^ For the sham experiment, the laser power was reduced close to zero (i.e., 0.1 W) with a black cap covering the laser aperture. In this way, the sham stimulation seemed similar to the actual TILS but without any light delivered to the subject’s forehead.

**Fig. 1:**
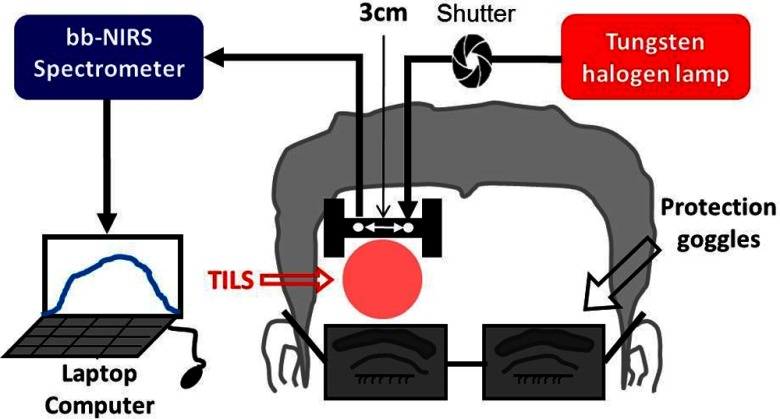
Schematic diagram of the experimental setup for TILS, including a bb-NIRS spectroscopic system. This bb-NIRS unit consisted of a tungsten-halogen lamp as the light source and a miniature CCD spectrometer as the detector. TILS was administered underneath the “I” shaped probe holder. The narrow, middle section of the holder was ∼8  mm in width. A laptop computer was used to acquire, display, and save the data from the spectrometer. A shutter controlled the on and off function for the white light from the tungsten–halogen lamp to the subject’s forehead. A pair of protection goggles was worn during the whole experimental procedure.^9^

Specifically, following previously successful studies,^4^^,^^5^^,^^10^^,^^13^ our safe laser stimulation parameters were as follows: total laser power=3.4  W; area of $\text{laser beam radiation}=13.6\;{\mathrm{cm}}^{2}$; $\text{power density}=3.4\;W/13.6\;{\mathrm{cm}}^{2}=0.25\;W/{\mathrm{cm}}^{2}$; time radiated per cycle=55  s; total laser energy per cycle=3.4  W×55  s=187  J/cycle; total laser energy density $\text{per cycle}=0.25\;W/{\mathrm{cm}}^{2}\times 55\;s=13.75\;J/{\mathrm{cm}}^{2}/\text{cycle}$. The TILS stimulation and measurement consisted of a 2-min baseline period, an 8-min laser stimulation period, followed by a 5-min recovery period. The stimulation site was on the right frontal forehead above the eye brow (see Fig. 1). Within each minute, the stimulation was on for 55 s and off for 5 s, when the bb-NIRS data acquisition was performed.

The experimental setup of a bb-NIRS system used for the previous TILS study is shown in Fig. 1; it was also utilized in the current heat-effect study. Section 2.3 below will provide detailed information on the bb-NIRS setup and related parameters chosen for bb-NIRS measurements.

### 2.2 Human Subjects Participated in TILS and Thermal Experiments

Eleven healthy human subjects were recruited from the local community of The University of Texas at Arlington (UTA) with 31±13.7 years of age (i.e., average±standard deviation) in TILS and thermal experiments. The two sets of experiments were carried out by two independent experimental designs with three visits (one visit for the TILS-induced effect and two visits for the heat-induced effects) of the same human participants. Prestudy screening was taken for each human participant during each visit prior to the stimulation/data acquisition. The inclusion criteria included either sex, any ethnic background, and in an age range of 18 to 50 years old. The exclusion criteria included: (1) diagnosed with a psychiatric disorder; (2) had history of a neurological condition, or any brain injury, or violent behavior; (3) had ever been institutionalized/imprisoned; (4) took any long-term or short-term medicine; (5) was currently pregnant; and (6) was a smoker or had diabetes.

TILS-induced metabolic and hemodynamic responses were measured and reported earlier.^9^ The current study focused on heat-induced changes of metabolic and hemodynamic signals on the forehead to determine whether thermal effects of TILS would potentially confound PBM effects that we observed previously.^9^ Specifically for the current thermal-effect study, the chosen individuals were assigned to participate in two separate experiments done on the forehead: (1) skin-temperature recording under TILS and (2) thermal stimulation done at the same forehead location as that of TILS, together with bb-NIRS measures as in our previous studies.^5^^,^^6^ These two experiments were performed during two separate visits. Specifically, skin-temperature-recording experiments were performed for all participants within 1 week. After the subject-averaged time-dependent temperature curve was acquired, all subjects underwent thermal stimulation experiments on their foreheads in the following week.

The experimental protocols adhered to National Institutes of Health (NIH) guidelines and were approved by the institutional review board (IRB) at UTA for ethical guidelines, which govern human experiments. Each participant received explanations of the instruments and procedures of the experiment. A written consent was taken from participants before the start of every experiment.

### 2.3 Experimental Setup and Instruments for Thermal Stimulation Measurements

**Fig. 2:**
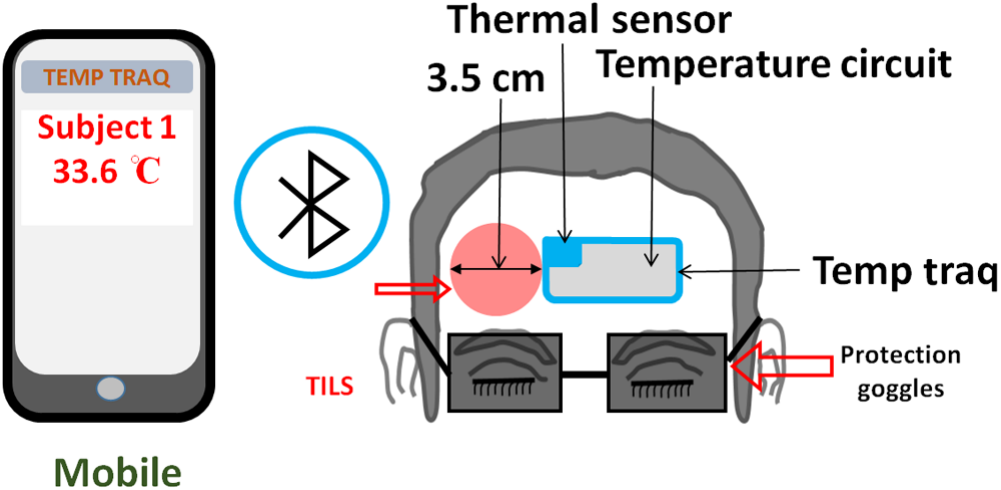
Schematic diagram of the experimental setup for the skin-temperature recording experiment that included a temperature measurement system (TempTraq™, Blue Spark Technologies, Inc., Westlake, Ohio). The TempTraq unit/patch includes a small thermal sensor ($5\times 5\;{\mathrm{mm}}^{2}$), as shown in the figure, and a temperature circuit to determine the temperature value induced by TILS on the forehead skin surface. TILS was delivered on the right side of the TempTraq patch, which was connected via Bluetooth to a mobile device. Protection goggles were worn by the participants during the entire experimental procedure.

The entire experiment was divided into two different phases. The first phase was to measure the skin temperature increase by the laser that was used in studies by Wang et al.^9^^,^^10^ As illustrated in Fig. 2, the laser used for the setup was a FDA-cleared 1064-nm laser device and also used before^9^^,^^10^ for laser stimulation. The uniform laser beam with an area of $13.6\;{\mathrm{cm}}^{2}$ was emitted from a safe distance of 2 cm from the handpiece during the experiment. Collimation of the laser facilitated the size of the laser beam at the stimulation area to be maintained as that emitted from the laser aperture. The laser power was maintained at a constant value of 3.4 W and laser power density in the laser beam was $0.25\;W/{\mathrm{cm}}^{2}$. The values of laser power and power density were chosen to replicate the experimental procedure conducted in previous studies^9^^,^^10^ so as to acquire the same TILS-induced temperature changes as before.

A thermal patch, TempTraq (hands-free temperature monitoring system) was used for measuring thermal readings near the TILS delivery site (right forehead) continuously during the entire experiment. This patch was made with safe, soft, flexible, durable, water-resistant, and nonlatex materials (TempTraq^™^, Blue Spark Technologies, Inc., Westlake, Ohio). In general, TempTraq is Bluetooth enabled to pair itself to any iOS or Android device for continuous monitoring of body temperature within a range of 40 feet. The patch was placed on the clean and dry skin surface; we ensured that no hair was trapped beneath the patch. The thermally sensitive area ($5\times 5\;{\mathrm{mm}}^{2}$) on the patch was located near the TILS location (marked by blue-shaded area in online Fig. 2). The rest of the patch included the embedded thermal detection circuit and the Bluetooth device (marked by gray-shaded area in online Fig. 2).

**Fig. 3:**
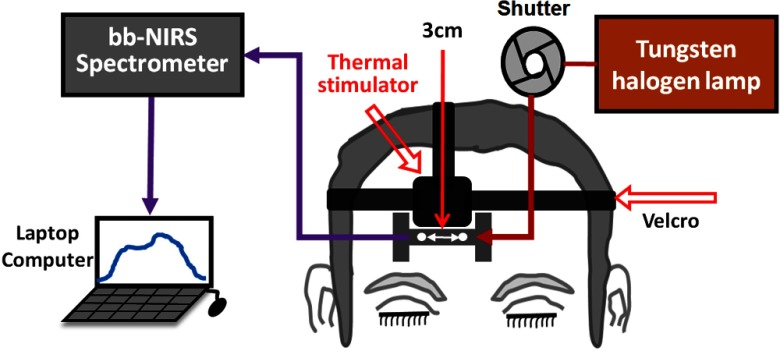
Schematic diagram of the experimental setup for the second phase of the experiment using a bb-NIRS monitoring system. The spectroscopic system consisted of a tungsten–halogen lamp as the light source and a high-sensitive CCD spectrometer as the detector. Thermal stimulation was administered above the I-shaped probe holder, which held two optodes with 3 cm apart. A shutter was used for switching the light delivery on and off from the lamp to the participant’s forehead. The data from the spectrometer were collected, saved, and displayed using a laptop computer.

The second phase of experiments was the thermal-stimulation measurements using a thermal stimulator (Pathway model ATS, Pain and Sensory Evaluation system, Medoc Advanced Medical Systems, Israel), which was employed to simulate TILS-induced thermal effects at the same location of TILS. The temperature output of the thermal stimulator was set according to the forehead skin-temperature experiment in response to TILS. The equipment has a dimension of 103  cm×52  cm×62  cm. The stimulation area of the ATS Thermode (probe) that came in contact with the skin surface was 16×16  mm. The temperature range that could be achieved was from 0°C to 55°C with an accuracy of 0.1°C. The rate of increase or decrease of the temperature could be programmed up to 8°C/s. As it is shown in Fig. 3, the ATS thermode of the Medoc pathway was placed on a clean surface of the right forehead for delivering thermal stimulation. The thermal stimulator was placed in close proximity of a 3D-printed, “I”-shaped, probe holder, which held the source and detector fibers to measure the metabolic and hemodynamic responses to thermal heating equivalent to that induced by TILS.

The bb-NIRS system consisted of a broadband light source (i.e., a tungsten-halogen lamp) having a spectral range of 400 to 1500 nm (Model 3900, Illumination Technologies Inc., East Syracuse, New York), a high-sensitivity CCD spectrometer with a spectral range of 735 to 1100 nm (QE-Pro, Ocean Optics Inc., Dunedin, Florida), and a laptop computer for data acquisition. Specifically, the light emitted from the light source was directed through a multimode fiber optode (diameter=3  mm) to the subject’s forehead. The diffused light from the forehead was collected by another fiber optode with the same size to the bb-NIRS spectrometer. The two fiber bundles were held by a 3D-printed I-shaped holder very closed to the stimulation site (see Fig. 3). Each acquired optical signal was sent to the QE-Pro spectrometer and then converted to a spectrum of 735 to 1100 nm for further spectroscopic analysis. The laptop computer would display and store the results for offline analysis and interpretation. Details of the bb-NIRS system can also be found in Refs. 9 and 10.

### 2.4 Experimental Protocols for Thermal Stimulation Measurements

#### 2.4.1 Forehead skin temperature recording in response to TILS

**Fig. 4:**
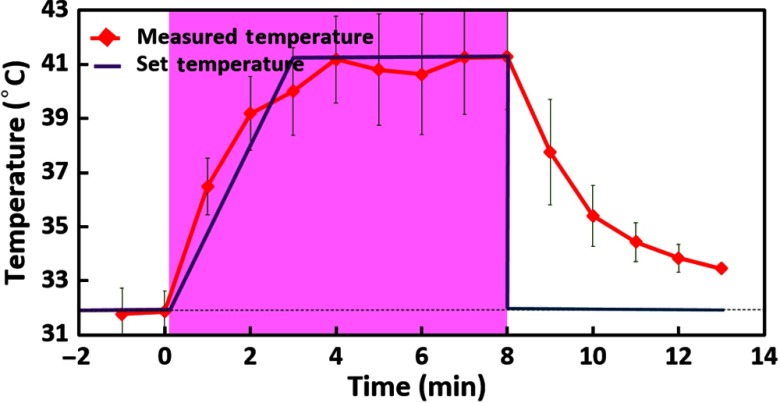
Forehead skin temperature increases during and post-TILS. It displays local skin temperatures of the forehead near the TILS site. The red curve with solid diamonds displays measured temperatures of the skin near the laser delivery site from the first phase (skin-temperature-recording) measurement. Each red solid diamond displays a single mean value with standard deviation, averaged over 1 min of the thermal data (also over n=11 participants). The blue lines without any symbols shows the thermal setting values on the thermal stimulator used in the second phase of the experiment. Time zero marks the starting time of TILS delivery.

During each experiment in both measurement phases, the subjects were comfortably seated, and procedures of the experiments were well explained. They were also asked to wear protective glasses for safety purpose and were instructed to close their eyes during the entire experimental procedure. The 1064-nm laser was used to stimulate the right forehead of each subject, and the laser hand piece was held by a well-trained research assistant to deliver TILS. A temperature sensor patch, TempTraq, was placed on the right forehead, close to the TILS site (see Fig. 2). The protocol and timeline used for TILS remained the same as those given in Refs. 9 and 10. In order to achieve accurate TILS-induced, skin-temperature readings, the thermal recording was on continuously during the entire TILS experiment. Then, time-dependent (averaged over 1 min) temperatures across the pre-, during-, and poststimulation period were calculated and are plotted in Fig. 4, outlining estimated skin temperatures near the light delivery site during and after TILS. This time-dependent, thermal profile then was used to set the temperature setting on the thermal stimulator (Medoc pathway) to simulate TILS-induced thermal effects. The blue (seen in online version) lines in Fig. 4 mark the thermal temperature setting that was used to create thermal stimulations in the second phase of the experiments, namely to measure metabolic and hemodynamic responses to thermal effects.

#### 2.4.2 Metabolic and hemodynamic responses to thermal stimulation

For the second phase of measurements, each participant was asked to relax without moving the head while reduced light scattering coefficient ($\mu_{s}^{\prime }$) and absorption coefficient ($\mu_{a}$) of the forehead were measured with a frequency-domain tissue oximeter (OxiplexTS, ISS Inc., Champaign, Illinois), as described in Refs. 9 and 10. After this set of optical property measurements, an “I”-shaped, optical probe holder was fixed on the right forehead near the TILS delivery site illuminated in the first phase (skin-temperature-recording) study (see Figs. 2 and 3). The distance between the source and detector fiber bundles in the holder remained 3 cm (see Figs. 1 and 3), the same as that in the first phase and an earlier study.^9^ The thermode that delivered thermal stimulation was placed in good contact with the skin of the right forehead, just right above the probe holder (see Fig. 3). A hospital-graded, double-sided tape together with Velcro stripes was also used to securely hold the thermode and the “I”-shaped holder in place to minimize motion artifacts.

**Fig. 5:**
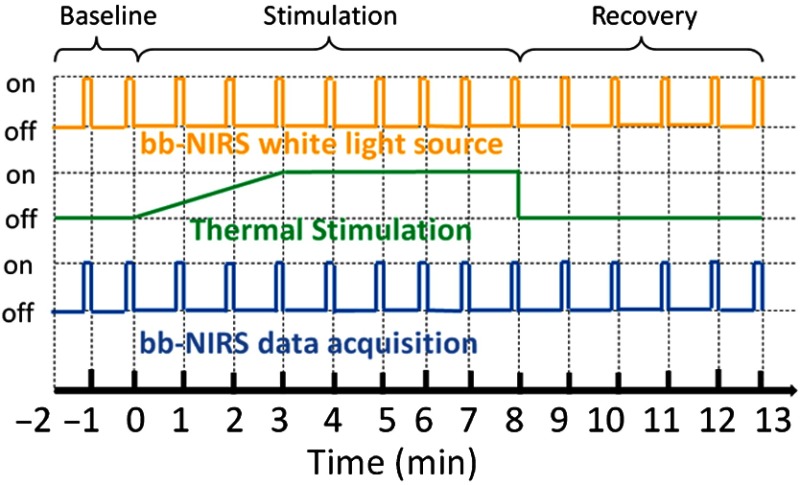
Experimental paradigm of bb-NIRS data acquisition pre-, during-, and postthermal stimulation delivered to the right forehead of each subject. Each experiment consisted of 2-min baseline, 8-min thermal stimulation, and 5-min recovery. The shutter for the light source was switched on for only 5 s during bb-NIRS data acquisition in each minute. The thermal stimulation consisted of a gradual increase in temperature from 32°C to 41°C during 3 min and a constant temperature at 41°C for 5 min.

The phase-two (i.e., thermal-stimulation measurements recording) experimental protocol is illustrated in Fig. 5. The thermal stimulation setting consisted of three different periods: baseline (prestimulation) for 2 min, thermal stimulation for 8 min, and recovery (poststimulation) for 5 min, which followed the protocol we used in phase-one measurements and previous studies.^9^^,^^10^ The white-light source for bb-NIRS was kept “on” during the entire experiment while the shutter was switched “on” only during the five-second data acquisition period and “off” for the rest of the experiment to avoid the confounding thermal effect caused by the bb-NIRS white light, as schematically shown in Fig. 5. The optical broadband spectra were acquired from the right forehead of each subject in the same format as that in our recent study.^9^ Also, potential heating caused by the white light during the measurement period was reduced by a short-pass optical filter, passing only wavelengths shorter than 1000 nm. The temperature used for thermal stimulation was initiated at 32°C and was varied according to the thermal variation pattern obtained in the first phase measurement, as marked by the blue lines in Fig. 4. Namely, the thermode temperature was set to increase steadily during the first 3 min to reach 41°C and then to maintain unchanged for 5 min as thermal stimulation, followed by a prompt stop to the baseline temperature, 32°C.

### 2.5 Data Processing and Statistical Analysis

Based on the modified Beer–Lambert law, a multilinear regression model was applied to the acquired spectral data for estimations of Δ[HbO], Δ[HHb], and Δ[CCO] in response to thermal effects. Mathematical details on both the modified Beer–Lambert law and multilinear regression model can be found in Refs. 9 and 10. Additionally, concentration changes of total hemoglobin (Δ[HbT]) and differential hemoglobin (Δ[HbD]) were estimated by Δ[HbT]=Δ[HbO]+Δ[HHb] and Δ[HbD]=Δ[HbO]−Δ[HHb], respectively, for all the 13 time points. The data for each time point across all 11 subjects were averaged. Also, the standard deviation and standard error of mean were computed. Statistical analysis was then carried out to determine statistically significant differences between the thermally induced and TILS-induced effects on Δ[HbO], Δ[HHb], Δ[HbT], Δ[HbD], and Δ[CCO] using a repeated-measure ANOVA, followed by one-way ANOVAs with the level of Bonferroni corrected significance of p<0.05 (to account for multiple-time measurements) in order to identify significant differences at individual time points, for each of the five chromophore concentrations.

## 3 Results

### 3.1 TILS-Induced, Time-Dependent Changes in Concentrations of CCO, HbO, HHb, HbT, and HbD

**Fig. 6:**
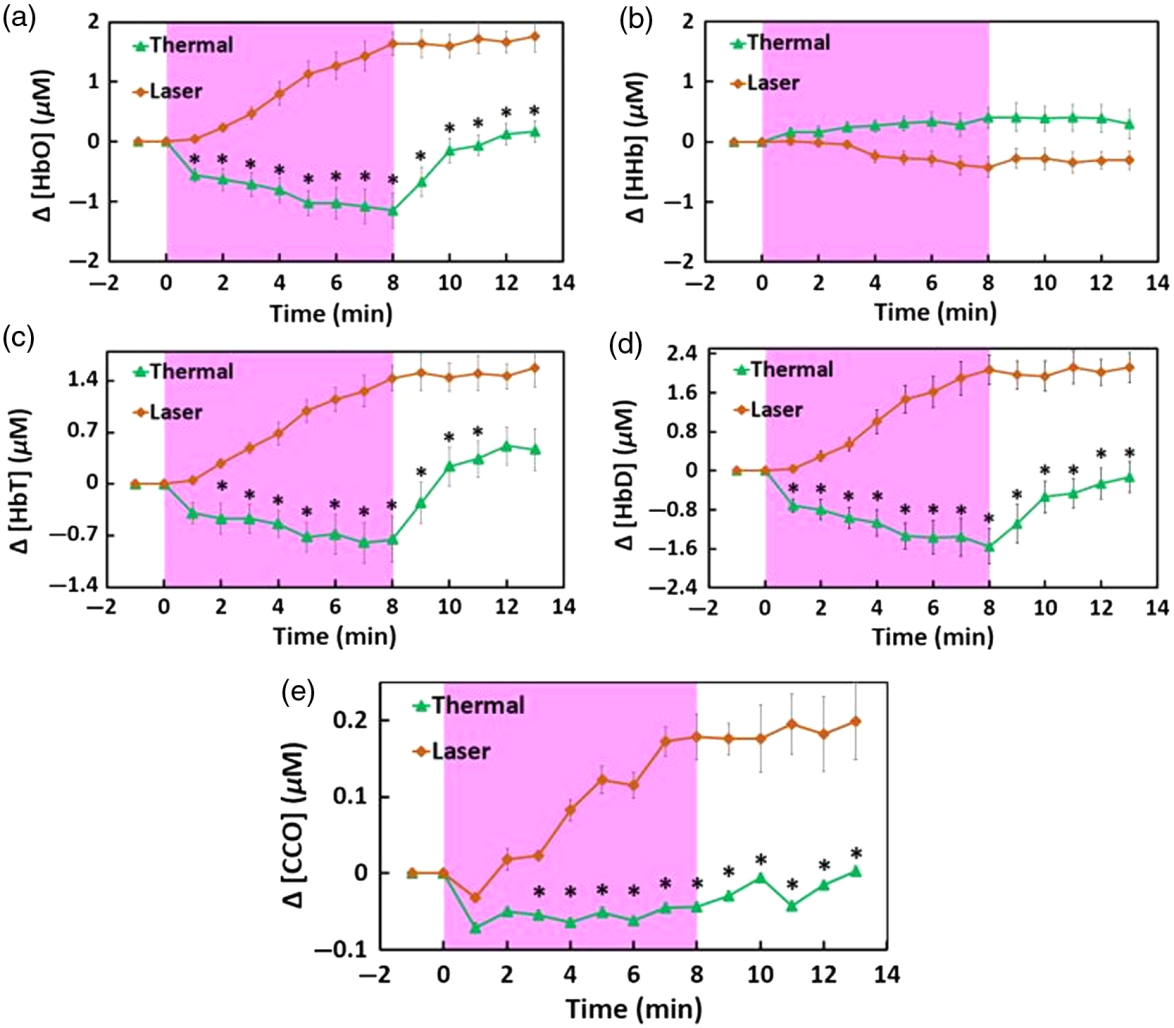
Participant–averaged time courses of TILS (laser) and heat stimulation (thermal) effects on changes in (a) [HbO], (b) [HHb], (c) [HbT], (d) [HbD], and (e) [CCO], measured *in vivo* from each participant’s forehead (mean±SE, n=11). The initial time at t=0 marked the onset of the TILS/thermal stimulation. The shaded region in each subplot displays the stimulation period. The unit for all concentration changes is in μM. In each panel, “*” symbols mark statistical significance with p<0.05 (Bonferroni corrected) between TILS-induced and heat-induced chromophore concentration changes, based on repeated measure ANOVA followed by one-way ANOVAs.

Figure 6 illustrates changes in concentrations of HbO, HHb, HbT, HbD, and CCO over the entire experiment of 15 min, including 2-min baseline, 8-min TILS/thermal stimulation, and 5-min recovery. Figure 6(a) displays two time-dependent curves of Δ[HbO] in response to TILS and thermal (heat) stimulation. Note that the TILS-induced Δ[HbO] values were reported previously in Ref. 9, but they are reused and replotted in this section for easy comparison. For each respective case, each data point was averaged over all the subjects; the shaded region indicates the time period under either TILS or thermal stimulation. The initial time at t=0 marked the onset of the TILS/thermal stimulation. Since the laser energy density (E) delivered to the forehead can be defined as a product of the exposure time ($t_{\text{exposure}}$) and the laser power density (P), namely, $E=t_{\text{exposure}}\times P$, the delivered stimulation dose is in proportion to the exposure time, as marked in Fig. 6. All five panels in Fig. 6 present dose–response curves, showing the dependence of metabolic/hemodynamic response parameters (i.e., Δ[CCO], Δ[HbO], Δ[HHb], Δ[HbT], and Δ[HbD]) on the TILS or heat stimulation dose over the entire experiment time course.

The repeated-measure ANOVA showed that either time or stimulation (i.e., thermal and laser) could create overall statistical significance for each of four chromophore concentration changes, namely, Δ[HbO], Δ[HbT], Δ[HbD] and Δ[CCO]. Next, one way ANOVAs with Bonferroni correction, performed at each individual time point for each of the chromophore concentrations, presented that significant differences between heat-induced and TILS-induced changes in [HbO], [HbD], [HbT], and [CCO] started to appear 1 min, 1 min, 2 min, and 3 min after the stimulation, respectively, as marked in each panel of Fig. 6. However, the repeated-measure ANOVA given on the time-dependent changes in [CCO] values showed that there was no significant difference in [CCO] changes with respect to that at 1 min after the heat stimulation. Based on the published work by Tsuji et al.,^19^^,^^20^ Soul et al.,^21^ and Hupert et al.,^22^ changes in [HbT] are directly linked to changes in tissue blood volume (ΔTBV) while changes in [HbD] are associated to changes in tissue blood flow (ΔTBF). Hence, Figs. 6(c) and 6(d) imply that the heat-based thermal stimulation on the human forehead resulted in significant changes in ΔTBV and ΔTBF during the thermal stimulation period.

Two other findings are worthwhile to point out: (1) all of the heat-induced hemodynamic decreases in Δ[HbO], Δ[HbD], and Δ[HbT] returned toward baseline within 2 to 3 min as soon as the termination of thermal heating while the TILS-induced increases in both hemodynamic and metabolic (i.e., Δ[CCO]) measures stayed constant, without any clear trend to quickly go back to baseline during the 5-min poststimulation period. (2) Figure 6(b) illustrates that heat-induced Δ[HHb] did not create any significant difference at any time point from those caused by TILS.

### 3.2 Dependence of Tissue Hemodynamic Parameters on Thermal-Induced Metabolic Changes

**Fig. 7:**
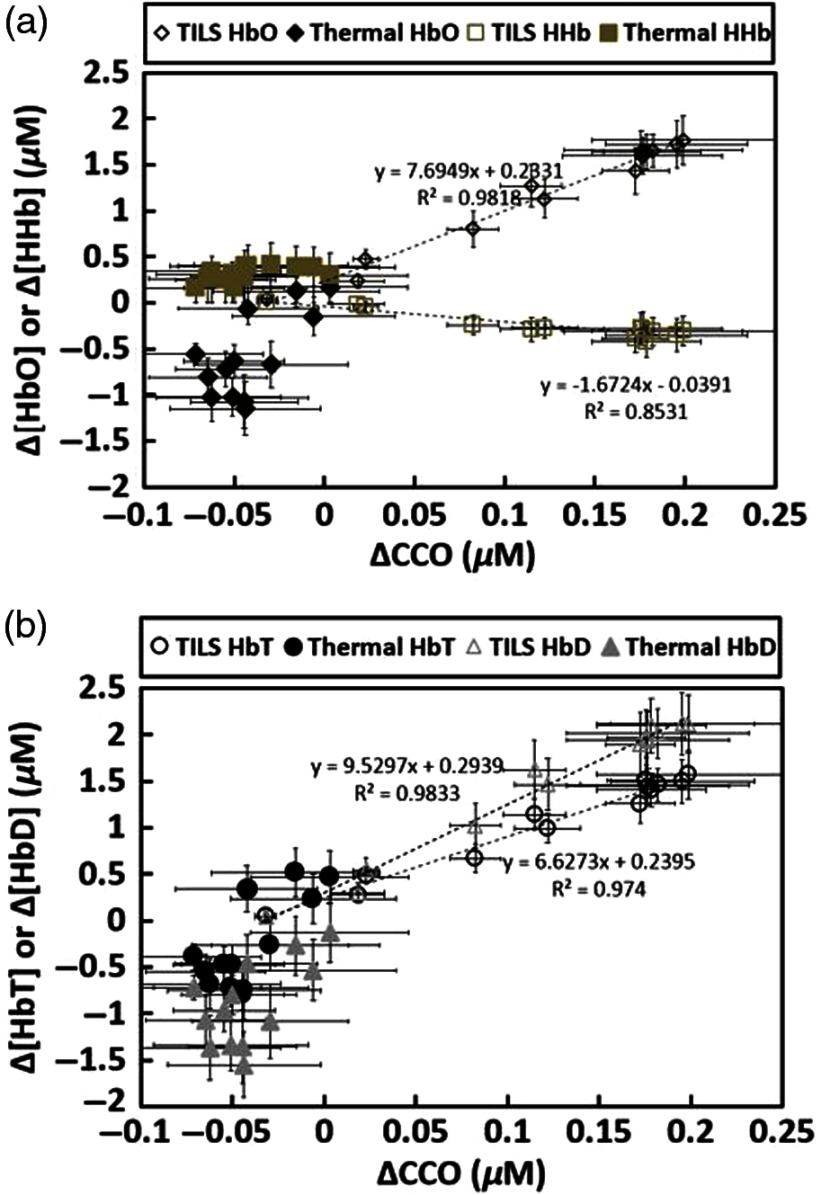
(a) Comparison of relationships between Δ[CCO] versus Δ[HbO] and Δ[CCO] versus Δ[HHb] across all subjects (n=11) under TILS and thermal stimulation. The solid black diamonds and solid tan squares show relationships of Δ[CCO] versus Δ[HbO] and Δ[CCO] versus Δ[HHb], respectively. Both open diamonds and squares symbolize TILS-induced Δ[CCO] versus Δ[HbO] and Δ[CCO] versus Δ[HHb], respectively. (b) Comparison of relationships between Δ[CCO] versus Δ[HbT] and Δ[CCO] versus Δ[HbD] across all subjects (n=11) under thermal stimulation and TILS. The solid black circles and solid gray triangles illustrate the relationship of Δ[CCO] versus Δ[HbT] and Δ[CCO] versus Δ[HbD], respectively. Both open circles and triangles denote the TILS-induced relationships. Error bars were based on standard errors of means for each respective chromophore concentration.

For better comparison, TILS- and heat-induced changes in [HbO], [HHb], [HbT], and [HbD] were extracted, regrouped, and replotted in Fig. 7 to investigate/reveal the dependence of each hemodynamic parameter (i.e., Δ[HbO], Δ[HHb], Δ[HbT], and Δ[HbD]) of forehead tissue on the metabolic indicator (i.e., Δ[CCO]) under the influence of TILS and/or thermal stimulation. The key observation is that distinct from the TILS case, no significant linear relationship existed between Δ[CCO] versus Δ[HbO], and Δ[CCO] versus Δ[HHb] under the thermal stimulation given on the forehead surface, although the heating/stimulation temperature followed exactly the thermal response to the TILS (determined by the first phase measurements). As shown clearly in Fig. 7(a), all Δ[HbO] and Δ[HHb] values during thermal stimulation lie within a lower range of Δ[CCO] (see the solid diamonds and squares, respectively, in the figure), as compared to a strong linear dependence of Δ[HbO] on Δ[CCO] under TILS (see the open diamonds in the figure). Figure 7(b) also exhibits similar trends of no clear dependence of Δ[HbT] and Δ[HbD] on Δ[CCO] (marked by solid circles and triangles, respectively), during the entire thermal stimulation period.

## 4 Discussion

### 4.1 Hemodynamic and Metabolic Responses of Forehead Tissue to Thermal Stimulation

In one of our recent studies, we clearly demonstrated that transcranial PBM by the 1064-nm laser gave rise to the upregulation of oxidized CCO concentrations and hemoglobin oxygenation *in vivo* assessed/quantified by noninvasive bb-NIRS.^9^ However, it was not clear whether the measured signals were contaminated by potential thermal effects that could result from possible TILS-related laser heating on the human forehead. To address this concern, we designed a novel protocol that utilized the same bb-NIRS to quantify thermal effects caused by the laser heating. In this way, we were able to assess heat-generated metabolic and hemodynamic parameters *in vivo* for the first time. To simulate the same thermal effects created by the 1064-nm laser, a thermal sensor was calibrated and used to facilitate time-dependent, skin-temperature recording near the TILS delivery site (Fig. 2). Then, a computer-controlled thermal stimulator was carefully set to deliver the same thermal variation pattern as that during TILS (Fig. 3). By comparing the chromophore concentration changes caused by both TILS (as reported in Ref. 9) and heat stimulation, we successfully demonstrated the distinction between heat-induced and TILS-induced responses in the hemodynamic and metabolic signals, which enabled us to exclude the potential confounding effect due to laser heating to the subject’s forehead.

Specifically, the experimental results shown in Fig. 6 clearly illustrated that transcranial Δ[HbO], Δ[HbT], and Δ[HbD] during the 8-min heat stimulation decreased significantly, implying reduced blood oxygenation, blood flow, and blood volume at the measured site. All three of the altered hemodynamic parameters returned promptly to baseline after the heat stimulation was removed. The statistical analysis, based on a repeated measure ANOVA followed by one-way ANOVAs, revealed that TILS resulted in strong hemodynamic oxygenation and metabolism, whereas heat applied to the forehead, on the other hand, generated cerebral hemodynamic effects distinct from those of TILS. Moreover, as shown in Fig. 7, no obvious linear interplay between hemodynamic and metabolic effects was observed during and after pure thermal stimulation.

While TILS-induced and thermally induced Δ[CCO] changes showed significant differences, the statistical analysis revealed that heat stimulation could not make Δ[CCO] significantly deviate from its initial onset value (i.e., 1 min after the heat stimulation) [Fig. 6(e)]. This observation implies that forehead thermal stimulation over 8 min up to 41°C did not significantly alter oxygen metabolism of forehead tissue, and thus would not significantly affect/confound our previous results and conclusions that TILS is able to upregulate CCO concentrations and hemoglobin oxygenation *in vivo* in human subjects.^9^ Another significant difference in forehead tissue responses to TILS and thermal stimulation is that both metabolic and hemodynamic changes during post-TILS tended to stay for a longer duration of time while these respective parameters returned quickly back to their baselines as soon as the heat stimulation stopped (Fig. 6). This observation supports the statement that TILS is highly desirable for treating certain neurological disorders because of its long-lasting after-effects.

The heat intervention was observed to generate hemodynamic and metabolic changes in the distinct direction or trend with respect to TILS. Thus, it is possible that the actual TILS-evoked changes in hemodynamic and metabolic enhancement could be greater than being reported in Ref. 9 if appropriate calibrations were taken to compensate for the laser-heating effect.

### 4.2 Possible Explanation of Heat-Induced Changes in Hemodynamic Signals of Forehead Tissue

In principle, thermal heating on a subject’s forehead should result in a temperature rise of forehead tissue, leading to the dilation of blood vessels and increase of regional blood flow at the stimulation site. This would also give rise to increases of total hemoglobin concentrations in the local stimulation site. However, the major temperature enhancement should happen only on the skin surface without affecting cerebral hemodynamics. Thus, any increase of tissue blood flow and blood volume (i.e., ΔTBF and ΔTBV) would occur only at the heating site, driving more blood from nearby superficial layers and resulting in decreases of ΔTBF and ΔTBV of nearby forehead tissue. Close inspection of Fig. 3 reveals that our bb-NIRS optodes interrogated a region of forehead tissue very adjacent to the heat-stimulation site. Thus, our observations on heat-induced hemodynamic changes shown in Fig. 6 match well the aforementioned expectation.

While a 3-cm source–detector separation of bb-NIRS could sense changes of hemodynamic signals in the cerebral regions, it measures all the signals coming from multiple layers below the two optodes, including the scalp, skull, and cerebral regions. It is noted that bb-NIRS detects only changes with respect to a baseline. Thus, it is expected that contributions from the superficial layers to the measured signals would become dominant if no or little change occured within the cerebral region. To confirm our expectation, a two-channel bb-NIRS system is needed with a short (1 cm) and long (>3  cm) source–detector separation in future studies, as pointed out in the following subsection.

### 4.3 Limitation of the Study and Future Work

First, this heat-stimulation study did not include a placebo experiment with respect to thermal stimulation, assuming that there were no variations in any of the NIRS parameters over the baseline readings. This assumption may not be accurate since cerebral hemodynamic signals (such as HbO, HHb, and HbT) do fluctuate over time. Moreover, both bb-NIRS and thermal probes placed on the subject’s forehead could give rise to time-dependent signal variations. All of these factors could confound the measured signals in the heat-stimulation group. In our future studies, we will conduct placebo controlled experiments in order to understand/reveal more rigorous/accurate thermal effects on hemodynamic and metabolic variations of forehead tissue.

Second, we were limited by the number of spectrometers (or channels) used in this study, so we could not perform two-channel (for long- and short-separation) broadband NIRS measurements to simultaneously monitor hemodynamic and metabolic changes at different tissue depths on the human forehead. Further upgrade on our instrumentation is needed for more comprehensive experiments to confirm our current findings.

## 5 Conclusion

In conclusion, we measured time-dependent temperature increases on 11 subjects’ foreheads using clinical-grade thermometers following the TILS experimental protocol used in our previous study. According to the broadband NIRS readings on the same subjects, significant differences in hemodynamic and metabolic responses (i.e., ΔHbO, ΔHbT, ΔHbD, and ΔCCO) were observed between the heat-induced and laser-induced effects on human foreheads. No obvious linear interplay between hemodynamic and metabolic effects was observed during and after pure thermal stimulation. The observations indicated that the tissue–heat interaction exhibited distinct response patterns from those during the tissue–laser interaction. This study overall demonstrated that the observed effects of TILS on cerebral hemodynamics and metabolism are not induced by heating the skin.
